# Rectal Aerophagia

**Published:** 1915-01

**Authors:** 


### ABSTRACTS

---

**Rectal Aerophagia.** Taillers, Arch. des Mal. de l'Appareil Digestiv Jan. 1914, describes a case in a girl of 14. She would assume the genu-pectoral position, relax the anus and allow air to enter and subsequently manifest conspicuous flatulence. The author states that the habit is common among soldiers, who subsequently compete for a prize given for the one who can blow out a candle at the greatest distance. There is a vulgar saying in English, perhaps in other languages, alluding figuratively to rectal aerophagia so that undoubtedly the habit has been common in various countries.

